# Sustainable Carbon Materials from Sucrose as Anodes for Sodium-Ion Batteries

**DOI:** 10.3390/molecules30051003

**Published:** 2025-02-21

**Authors:** Belén Lobato, Nuria Cuesta, Ignacio Cameán, Maria Rosa Martínez-Tarazona, Roberto García, Ana Arenillas, Ana B. García

**Affiliations:** Instituto de Ciencia y Tecnología del Carbono—Consejo Superior de Investigaciones Científicas (INCAR-CSIC), Francisco Pintado Fe 26, 33011 Oviedo, Spain; n.cuesta@incar.csic.es (N.C.); icamean@incar.csic.es (I.C.); robo@incar.csic.es (R.G.); aapuente@incar.csic.es (A.A.); anabgs@incar.csic.es (A.B.G.)

**Keywords:** sucrose, sustainable carbon materials, anodes, sodium-ion batteries

## Abstract

The implementation of sodium-ion batteries for renewable energy storage requires the development of sustainable electrode materials. Usually, these materials are produced through complex energy-intensive processes that are challenging to scale and involve expensive and/or toxic reagents. In this study, sustainable hard carbon materials, some doped with iron, synthesized from sucrose using a simple, fast, and cost-effective two-step eco-friendly process, are investigated as anodes for sodium-ion batteries. The influence of physicochemical and structural material properties on electrode reversible capacity, cycling stability, and efficiency is analyzed. The SC900 material, which exhibits a certain development of graphite-like structure, though not strictly graphitic, showed the best electrochemical performance, providing discharge capacities exceeding 100 mAh g^−1^ after 400 cycles with excellent cycling stability and high coulombic efficiency. The capacity of the materials increases as d_002_ decreases, (i.e., as the degree of structural order increases), to the optimum value of ~0.3700 nm. However, a further decrease in d_002_ to values characteristic of quasi-graphitic materials, as a consequence of the catalytic effect of iron, hinders Na^+^-ion storage, which, in addition to the low electrochemical activity of the iron oxides present, leads to much lower capacities.

## 1. Introduction

The implementation of electricity generation from renewable energy sources is essential to ensuring global access to affordable, reliable, sustainable, and modern energy, aligning with the 7th Sustainable Development Goal adopted by all United Nations Members in 2015 [1]. However, the intermittent and stochastic nature of these energy sources requires the development and optimization of cost-effective and eco-friendly energy storage systems. In this context, sodium-based energy storage, particularly sodium-ion batteries (SIBs), offers significant advantages, including sodium’s great natural abundance (ranked as the sixth most abundant element) and widespread global distribution, which contribute to lower costs. Additionally, sodium exhibits lower toxicity compared to other battery materials. Another key advantage of SIBs is the ability to use aluminum as a current collector for both electrodes, significantly reducing production costs. For the implementation of medium- and large-scale renewable energy storage systems, economic considerations are essential, often taking precedence over technical performance [2,3,4,5]. The considerable interest shown by the scientific community in SIBs has driven the development and identification of various suitable cathodes, electrolytes, and anodes [2,5]. For the latter, carbon and carbon-based materials have been extensively studied [6,7,8], with the exception of graphite, which has limited Na^+^ ion intercalation capability [9]. These materials exhibit significant advantages, including a wide range of precursors, high availability, and cost-effectiveness. Among them, hard carbons (non-graphitizable) have been extensively studied and identified as the most suitable anode materials for SIBs [10,11,12], as they can be obtained from fossil fuels, biomass, or polymeric carbons by multiple synthetic routes, resulting in a wide variety of scalable and cost-effective materials with a combination of unique and tailorable properties.

In recent years, interest in developing sustainable biomass-derived hard carbon materials for sodium-ion battery electrodes have grown enormously [13,14,15,16]. From a sustainability and availability point of view, the use of renewable sources such as biomass is highly appealing. However, the reproducibility of the carbon material properties to comply with high quality battery demands, specifically in terms of impurity control, remains challenging due to the variability in biomass origin and composition. Therefore, identifying sustainable and abundant precursors capable of yielding reproducible carbons through simple, cost-effective, and easily scalable processes is an essential step toward the widespread implementation of sodium-ion batteries as energy storage systems. In this context, sucrose, a highly abundant disaccharide containing 42.1 wt.% carbon, can be converted into hard carbon via dehydration followed by heat treatment [17]. Sucrose-based hard carbons were first investigated as sodium-ion battery anodes by Stevens and Dahn, at very low current densities, yielding moderate performance results [17]. More recently, various methodologies have been explored to optimize the porosity of these sustainable materials and enhance their electrochemical performance [18,19,20,21,22,23,24,25]. Typically, sucrose-derived hard carbons are produced by microwave irradiation or hydrothermal reaction, followed by pyrolysis treatment at temperatures in the 800–2000 °C range for several hours. Nagmani et al. [22,23] and Pan et al. [21] prepared sucrose-based hard carbon microspheres by microwave-assisted solvothermal pre-treatment followed by carbonization. Moreover, the latter used acetonitrile chemical vapor deposition to fill micropores, thereby reducing the specific surface area of the microspheres. Zhang et al. prepared low-surface-area hard carbon from a mixture of sucrose and phenol formaldehyde resin by pyrolysis at temperatures in the 1000–1600 °C range [24], whereas Luo et al. reported similar results by pyrolysis of sucrose and graphene oxide mixture [25]. These approaches can enhance certain properties of hard carbons, thereby improving their electrochemical performance. However, they often imply complex processes that might require significant power supply, pose scalability challenges, and, in some cases, involve the use of expensive and/or toxic reactants.

In this study, sustainable hard carbons, some doped with iron, were prepared from sucrose by a simple, fast, and cost-effective two-step process that involves minimal and non-toxic reagents. These carbons were investigated as active anode materials for sodium-ion batteries through prolonged galvanostatic cycling. The study examines the influence of the active material physicochemical and structural properties on electrode reversible capacity, cycling stability, and efficiency. Additionally, the mechanism of Na^+^ ion electrochemical storage in these materials is discussed.

## 2. Results and Discussion

### 2.1. Sucrose-Based Carbon Materials: Chemical, Textural, Structural, and Morphological Properties

The elemental analysis results of sucrose-based carbon materials, synthesized according to the procedure described in the Materials and Methods section, are presented in Table 1. Among the SCs, those prepared at carbonization temperatures in the 800–900 °C range (SC800, SC900) show comparable chemical compositions, with carbon, oxygen, and hydrogen contents of 95–96 wt.%, 2–3 wt.%, and ~1 wt.%, respectively. In contrast, lowering the carbonization temperature to 500 °C results in a carbon material (SC500) with significantly higher oxygen (~12 wt.%) and hydrogen (~3 wt.%) contents. As expected, the iron-doped sucrose-based carbon materials (2FeSC900, 9FeSC900) contain lower amounts of C, H, and O than undoped SC900. However, it is noteworthy that the decrease of these elements contents as a consequence of iron incorporation is significantly more pronounced for hydrogen and oxygen than for carbon. Specifically, in 9FeSC900, the carbon, hydrogen, and oxygen contents decrease by approximately 6%, ~71%, and 61%, respectively, compared to SC900.

The textural parameters of the materials, calculated from the corresponding N_2_ adsorption–desorption isotherms (Figure S1), are reported in Table 2. According to the IUPAC classification [26], FeSC materials present a type IV isotherm, with a hysteresis loop, due to the presence of mesopores, while SC materials do not show significant micro- or mesoporosity. 2FeSC900 and 9FeSC900 have moderate S_BET_ values of 163 and 195 m^2^ g^−1^, respectively, due to the presence of microporosity. However, they show a significant development of mesopores, especially in the material with higher Fe content. In essence, as concluded in previous work [27], iron doping in sucrose-based carbons promotes porosity formation, especially mesopores. While the amount of Fe introduced does not affect the mesopore size, it significantly influences the volume of this type of porosity. On the other hand, the external surface area (S_ext_), which is associated with the most accessible surface of the material, and closely related to diffusional processes, was also calculated for FeSC materials. S_ext_ also increases with higher amount of Fe used in the synthesis of the materials, due to the enhanced development of feeder pores (i.e., mesopores), making the most of the surface available with little diffusional restrictions.

**Figure 1:**
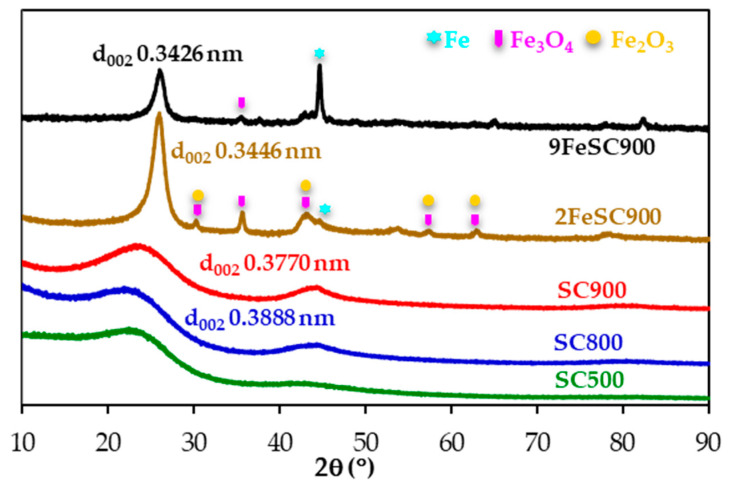
XRD patterns of SC and FeSC materials.

Figure 1 shows the X-ray diffractograms of the sucrose-based carbon materials. Increasing the carbonization temperature and, especially, doping with iron leads to sharper and more well-defined peaks, specifically that corresponding to the (002) reflection of the graphitic carbon. Moreover, the (002) peak maximum slightly shifts to higher 2θ values, due to the increased graphite-like structural order of these carbon materials, as evidenced by the gradual decrease of the interlayer distance, d_002_, calculated from the position of this peak by applying Braggs’s equation. Therefore, 9FeSC900, prepared at 900 °C in the presence of iron species, which are known to be excellent graphitization catalyst of sustainable carbon precursors [28], presents the lowest d_002_ value (0.3426 nm), followed by 2FeSC900 (d_002_ = 0.3446 nm). Based on the classification of Rosalind Franklin [29], graphitic materials are those with d_002_ values in the 0.3354–0.3400 nm range. Accordingly, although FeSC materials in this work do not strictly qualify as graphitic, it is obvious that they have a significant degree of three-dimensional structural order, suggesting that they can be considered quasi-graphitic. In contrast, SC900 and SC800 exhibit d_002_ values, in the range of 0.3770–0.3888 nm, characteristic of carbon materials with small graphitic domains, while the (002) peak of the material carbonized at the lowest temperature (SC500) is too broad for d_002_ calculation. Moreover, peaks corresponding to magnetite (Fe_3_O_4_) and maghemite (Fe_2_O_3_) are clearly detected in the XRD pattern of 2FeSC900. As the amount of iron increases in (9FeSC900), maghemite peaks disappear, leaving only small magnetite peaks and a strong peak at 44.6°, attributed to metallic iron (Figure 1).

**Figure 2:**
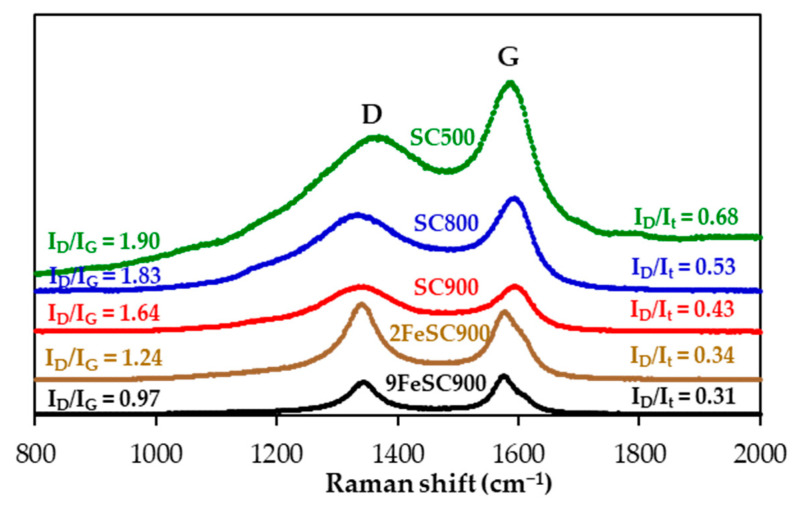
First-order Raman spectra of SC and FeSC materials.

Raman spectroscopy was employed to conduct a more detailed analysis of the surface structural defects of the materials. As illustrated in Figure 2, the first-order spectra of the SC and FeSC materials reveal broad bands at approximately 1580 cm^−1^ and 1350 cm^−1^, corresponding to the G-band and D-band, respectively. The G-band is associated with the in-plane bond stretching motion of crystalline graphite, while the D-band corresponds to defects or disorder in the graphitic structure. The observed decrease in the relative intensities of the D-band, I_D_/I_t_ and I_D_/I_G_, and in the widths of the G-band (W_G_) and D-band (W_D_) as the carbonization temperature increases and/or with iron doping, which indicate the development of a more ordered graphitic structure, corroborates the XRD data discussed above.

**Figure 3:**
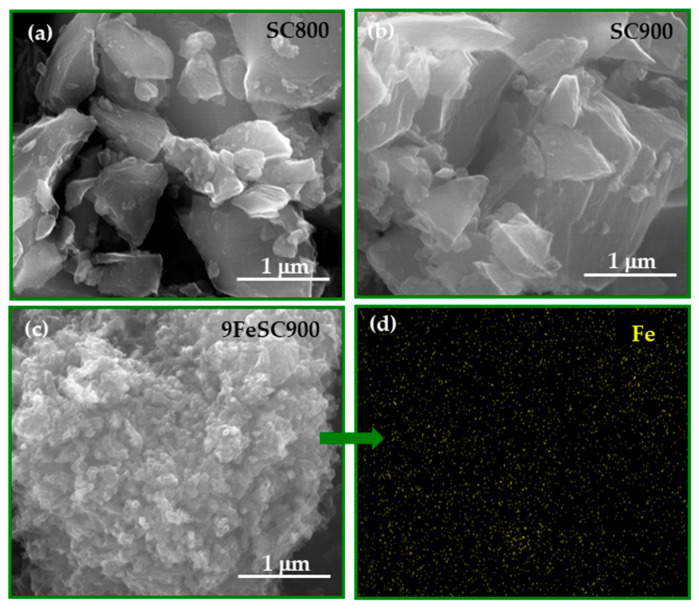
SEM images at 80,000× of SC800 (**a**), SC900 (**b**), and 9FeSC900 (**c**), and EDX image of 9FeSC900 (**d**).

Figure 3 shows the SEM micrographs of SC800, SC900, and the iron-doped 9FeSC900 as representative examples of the sucrose-based carbon materials prepared. As observed, the increase of SC carbonization temperature (from SC800 to SC900) leads to the formation of stacks of quasi-parallel graphene layers, which agrees with the increase in the graphite-like structural order of the material, as indicated by the decrease in the d_002_ interlayer spacing (Figure 1). On the other hand, doping with iron induces a significant change in the morphology of the sample, resulting in a much rougher surface for 9FeSC900. Furthermore, the EDX analysis confirms the homogeneous distribution of the iron throughout the material.

At this point, it is important to mention that: (i) theoretical calculations indicate that for the electrochemical storage of the Na^+^ ions in carbon materials by a reversible intercalation/insertion mechanism, an optimum d_002_ of ~0.3700 nm is required, as it is associated with a certain degree of structural order, i.e., the presence of small graphitic domains [30], but not strictly graphitic materials (0.3354–0.3400 nm), which hardly store Na^+^ ions by this mechanism due to thermodynamics reasons [9], and (ii) the presence of iron/iron species in carbon materials is known to improve the storage of Na^+^ ions during the electrochemical process [31]. Therefore, these two factors should be considered when discussing the performance of SC and FeSC materials as anodes for sodium-ion batteries.

### 2.2. Sucrose-Based Carbon Materials: Performance as Anodes in Sodium-Ion Batteries

**Figure 4:**
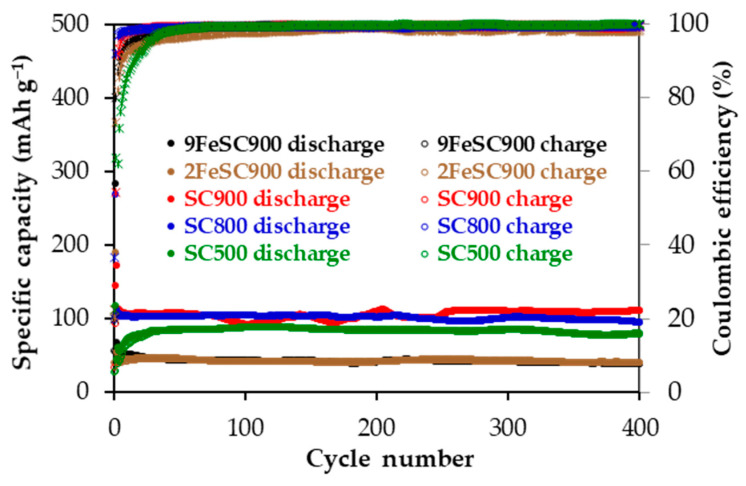
Specific capacity and coulombic efficiency as a function of cycle number for SC- and FeSC-based electrodes at 37.2 mA g^−1^ in the 0.01–2.10 V voltage range.

Table 3 compiles the main electrochemical parameters obtained from the galvanostatic cycling tests in the 0.01–2.10 V voltage range at an electric current density of 37.2 mA g^−1^ for SC and FeSC materials. Specifically, it presents the discharge capacities (C_disc_) at the 1st, 2nd, 25th, 100th, 200th, and 400th cycles, the irreversible capacity in the 1st cycle (C_irr_), and the capacity retention (R). SC-based electrodes provide C_disc_ above 80 mAh g^−1^ over 400 cycles, with excellent coulombic efficiency. Moreover, as shown in Figure 4, which plots the specific reversible capacity and coulombic efficiency versus cycle number, all of them exhibit remarkable cycling stability, particularly after cycle 25. At this point, capacity loss due to SEI layer formation and any other parasitic formation reactions occurring at low current density, as well as potential Na^+^ ion trapping (i.e., irreversibly insertion), appears to be almost over [32]. In any case, they account for the irreversible capacity values determined for SCs (Table 3). Among SCs, SC900 shows the best overall anodic performance, delivering a C_disc_ of 110 mAh g^−1^ at the end of cycling, with a coulombic efficiency of 99%, and a R of 99% between cycles 25 and 400. Slightly lower discharge capacities of 95 and 81 mAh g^−1^ were finally supplied by SC800 and SC500, respectively, with R values in the 94–93% range.

For comparison, similar capacity values (~100 mAh g^−1^ at 37.2 mA g^−1^ after 23 cycles) have been reported for other sucrose-based carbons also prepared by pyrolysis but under high pressure (up to 7·10^5^ N m^−2^) [18]. Stevens and Dahn [17] observed higher capacities for these sustainable materials, but only for the first cycle and at a very low current density (35 μA cm^−2^ vs. 132 μA cm^−2^ in this study). In addition, their material production process involved higher pyrolysis temperatures (1000–1150 °C).

Based on the characterization results discussed in Section 2.1, the improvement in the electrochemical performance of SC materials appears to be linked to the increase in their graphite-like degree of structural order (i.e., decrease of d_002_ and I_D_/I_t_ parameters). To gain further insight into this issue, the potential profiles (potential vs. Na/Na^+^ against capacity) from the galvanostatic cycling of SC materials for cycles 1, 2, 25, 50, 100, 200, and 400, shown in Figure S2, were analyzed in detail. First of all, the voltage profile shapes of SC500, SC800, and SC900 are similar; indicating that Na^+^ ion storage in these materials occurs through the same mechanism. During the first sodiation (discharge curve), three distinct potential regions can be identified in all materials: (I) a sharp sloping curve above ~0.7 V, (II) a more or less sloping plateau in the ~0.6–0.3 V range, and (III) a sloping curve below 0.3 V. In subsequent cycles, particularly after cycle 25 (capacity stabilization, Figure 3), the potential profiles only exhibit a sloping region below ~1 V, and, as expected, they nearly overlap. These differences are attributed to the above mentioned SEI formation [32]. Moreover, unlike other disordered carbon materials [33], no plateau region below 0.2–0.1 V is observed in the potential profiles of SC materials, suggesting that the storage of Na^+^ ions takes place predominantly through a single mechanism.

Despite extensive research aimed at understanding the mechanisms of electrochemical sodium ion storage in non-strictly graphitic carbons, this topic remains a subject of debate due to the complex microstructure of these materials [30,34,35,36,37]. Nonetheless, it is generally accepted that the Na^+^ ion storage primarily occurs through two mechanisms: (1) insertion/intercalation between the aromatic carbon layers of small graphitic domains and (2) adsorption on surface (defects and pores) [37,38,39]. Both processes are associated to the sloping region of the potential profiles, the first in the model proposed by Stevens and Dahn [17,39] and the second in the so-called adsorption–intercalation model [30]. Given that SC materials exhibit no porosity (Table 2), their capacity can be mainly attributed to the reversible insertion/intercalation of Na^+^ ions into graphene layers. Therefore, the increase in graphite-like structural order with higher carbonization temperatures (from 500 to 900 °C), reaching the optimal d_002_ of ~0.3700 nm, is responsible for the observed capacity enhancement discussed above.

**Figure 5:**
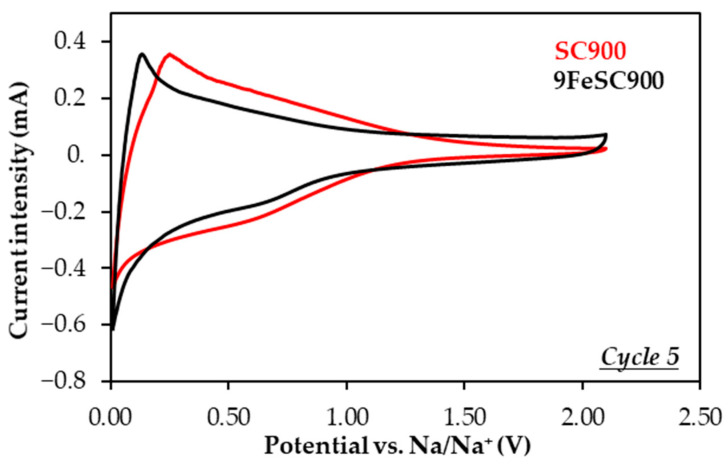
Cyclic voltammograms of SC900- and 9FeSC900-based electrodes at the 5th cycle, recorded at a scan rate of 0.2 mV s^−1^.

The doping of sucrose-based carbons with iron results in a significant decrease in material capacity (Table 3, Figure 4). For comparison, SC900 and 9FeSC900 exhibit discharge capacities of 111 and only 39 mAh g^−1^ at the end of cycling, respectively, with corresponding coulombic efficiencies of 99.3 and 99.8%. This reduction in capacity is also accompanied by a moderate decrease in capacity retention. The poor electrochemical performance of FeSC materials could be attributed to either their quasi-graphitic nature (Figure 1), which hinders Na^+^ ion insertion/intercalation [9], and/or the low electrochemical activity of the iron species present. To investigate this further, a comparative analysis of the cyclic voltammograms (CVs) of SC and FeSC materials was performed. As an example, Figure 5 shows the CV curves of SC900 and 9FeSC900 at cycle 5, recorded at a scan rate of 0.2 mV s^−1^. Both materials exhibit a strong cathodic (reduction) peak centered around 0.01 V, which corresponds to the sodiation of carbon. In the case of iron-doped carbons, this peak is also associated with the conversion of Fe^2+^/Fe^3+^ into Fe^0^ (reduction of iron oxides) and the formation of Na_2_O. Moreover, in the CV of 9FeSC900, a weak broad peak between 1.10 and 0.40 V is observed, corresponding to Na^+^ ion insertion into Fe_2_O_3_ to form Na_x_Fe_2_O_3_, along with the reduction of Fe^3+^ to Fe^2+^. During the anodic process of this iron-doped material, only a broad band appears in the ~0.08–1.50 V range, with a maximum centered at approximately 0.20 V, likely encompassing the typically weak and broad oxidation peaks at 0.5–2.0 V, which correspond to the regeneration of iron oxides. However, the characteristic sharp peak at ~0.08 V, assigned to Na^+^-ion de-insertion from iron-doped carbons [31,40] is absent. This confirms that Na^+^ ions do not insert into the aromatics layers of FeSC graphitic domains. Therefore, the capacity of iron-doped carbons appears to be mainly due to the electrochemical activity of the iron oxides present in these materials (Figure 1), without ruling out the possible contribution of microporosity, i.e., surface defects, which, according to the adsorption–intercalation model [30], can be associated with the sloping voltage profiles observed for these materials (Figure S2).

## 3. Materials and Methods

### 3.1. Synthesis of Sucrose-Based Carbons

Sucrose was used as the precursor for carbon synthesis, following a procedure previously described [27,41]. Briefly, sucrose was dissolved in a 0.01 wt.% citric acid aqueous solution. Citric acid serves as a catalyst in the caramel-forming hydrolysis of sucrose, which is carried out by heating the solution in air to 170 °C. The resulting caramel was further heated at a rate of 2 °C min^−1^ from room temperature to 240 °C in air, maintaining this temperature for 20 h, to yield a green carbon intermediate. After cooling, the green carbon was carbonized under argon stream (50 cm^3^ min^−1^) by heating at 4 °C min^−1^ to a final temperature of 500, 800, or 900 °C, which was held for 2 h. The resulting sucrose-derived carbons (SCs) were designated as SC500, SC800, and SC900. Following a similar approach, two iron-doped carbon foams were synthesized by initially adding Fe(NO_3_)_3_ · 9H_2_O to sucrose in weight ratios of 1:5 and 1:20. As iron nitrate also catalyzes sucrose hydrolysis, caramel (more resin-like in this case) formation occurred at 80 °C, and green carbon formation required only 170 °C. These Fe-loaded green carbons were exclusively carbonized at 900 °C. The iron content in the final carbon materials was determined after acid digestion of 50 mg in HCl:HNO_3_ (3:1 *v*:*v*), in a microwave oven, followed by solution analysis via ICP-MS (Agilent Technologies, Santa Clara, CA, USA. Iron contents of 8.80 and 2.20 wt.% were determined for these doped sucrose-based carbons, which were named as 9FeSC900 and 2FeSC900, respectively.

### 3.2. Characterization Techniques

The physicochemical properties of the materials, including chemical composition and textural characteristics, were determined using various analytical techniques. Elemental analysis of carbon, nitrogen, and hydrogen was conducted using a LECO CHN2000 instrument, whereas oxygen and sulfur contents were quantified with a LECO TruSpec_Micro-O and a LECO S632 (LECO Corporation, St. Joseph, MI, USA), respectively. Textural properties were assessed via helium pycnometry (AccuPyc 1330, Micromeritics, Norcross, GA, USA), and N_2_ adsorption–desorption isotherms at −196 °C (Tristar II, Micromeritics, Norcross, GA, USA). Prior to characterization, the materials were subjected to overnight vacuum outgassing at 120 °C. The Brunauer–Emmett–Teller (BET) method [42], Dubinin–Radushkevich (DR) equation [43], and t-plot equations [44] were applied to the adsorption branch of the nitrogen isotherms to calculate the BET surface area (S_BET_), micropore volume (V_micro_), and external surface area (S_ext_). The total pore volume (V_p_) was determined from the amount of nitrogen adsorbed at the saturation point (*p*/*p*_0_ = 0.99), while the mean pore size was determined using the 2D-NLDFT model.

The structural properties of the materials were investigated by X-ray diffraction (XRD) and Raman spectroscopy. XRD patterns were recorded on a Bruker D8 Advance instrument (Bruker Corporation, Billerica, MA, USA), operating at 40 kV and 40 mA with a Cu-Kα (λ = 0.15406 nm) radiation source. Data were collected over 2θ range of 10–90° (once) and 10–40° (twice) with a step size of 0.02° and an interval of 3 s per step, as described elsewhere [45]. A silicon standard was used to correct the broadening of the diffraction peaks caused by instrumental factors. The interlayer distance (d_002_) was calculated from the position of the (002) peak in the XRD pattern using Bragg’s equation, with standard errors below 0.3%.

Raman spectra were collected using Renishaw inVia™ Qontor confocal Raman spectrometer with a DPSS laser source operating at a wavelength of 532 nm and 10% power (Renishaw, Gloucestershire, UK). Each spectrum was recorded over the 800–2000 cm^−1^ range (2 accumulations of 10 s) and processed with WiRE 5.4 software. The widths, D, and the intensities, I, of the Raman bands were measured using a mixed Gaussian–Lorentzian curve-fitting procedure. The relative intensities of the Raman D-band, I_D_/I_t_ (I_t_ = I_D_ + I_G_ + I_D’_), and I_D_/I_G_ were calculated with standard errors below 0.01%.

The morphology of the materials was examined using scanning electron microscopy (SEM) with a Quanta FEG 650 (FEI) instrument (FEI Company, Hillsboro, OR, USA), operating at an accelerating voltage of 20 kV and equipped with an Everhart–Thornley secondary electron detector (ETD) and energy-dispersive X-ray spectroscopy (EDX). Samples were mounted on aluminum stubs using conductive double-sided adhesive carbon tape, with no additional coating applied.

### 3.3. Electrode Preparation, Cell Assembly, and Electrochemical Measurements

Electrochemical measurements were carried out using two-electrode Swagelok-type cells, consisting of a working electrode, WE, and a counter electrode, CE. The WEs were composed of 82 wt.% SC or FeSC (active material), 10 wt.% of carbon black (Super C65, Imerys Graphite & Carbon, Paris, France) as a conductive additive, and 8 wt.% of sodium carboxymethylcellulose (NaCMC, Sigma-Aldrich, Darmstadt, Germany, M_w_ ~700,000) as a binder. The electrode preparation involved the following steps: (i) NaCMC was dissolved in 20 mL of deionized water under mechanical stirring (IKA Overhead Stirrer Eurostar20, Barcelona, Spain), at 3000 rpm for 30 min, to obtain a 1.0 wt.% solution; (ii) carbon black was added to the NaCMC solution, while stirring at 1000 rpm for 10 min to ensure a homogeneous dispersion; (iii) the active material was gradually incorporated into the mixture to form a slurry, which was stirred at 4000 rpm for 1 h to prevent agglomeration; (iv) the slurry was tape-cast onto aluminum foil (99.3 wt.% purity, 20 µm thickness, Hohsen Corporation, Osaka, Japan) at 50 °C, using a standard doctor blade with a 250 µm gap and an Elcometer 4340 motorized film applicator equipped with a perforated heated vacuum table (Elcometer, La Chapelle Saint Mesmin, France); (v) the electrode tape was kept on the applicator table under vacuum at 80 °C for 1 h; (vi) circular electrodes (12 mm diameter) were punched from the tape using a manual punching machine; and (vii) the electrodes underwent an additional drying step at 120 °C for 2 h, under vacuum, in a Büchi B-585 glass oven (Büchi, Gallen, Switzerland), and were subsequently stored in an MBraun glove box (MBraun, Bayern, Germany), under an argon atmosphere, with oxygen and water contents below 0.1 ppm. The average active mass loading of the electrodes ranged from 2.7 to 4.2 mg cm^−2^.

Metallic sodium discs (≥99.9 wt.% purity, Merck/Sigma Aldrich, Darmstadt, Germany) with a diameter of 12 mm were employed as CEs. As separators, two 12 mm diameter microfiber glass discs (WHATMAN, Buckinghamshire, UK), soaked with 250 µL of the electrolyte, 1.2 M solution of sodium hexafluorophosphate (NaPF_6_, >99% purity, Sigma Aldrich, Darmstadt, Germany) in a 1:1 (w:w) mixture of ethylene carbonate, EC (99.0% purity, Merck, Darmstadt, Germany), and ethyl methyl carbonate, EMC (≥99.9% purity, Sigma-Aldrich, Darmstadt, Germany), were placed between WE and CE. The cell assembly was performed inside the glove box, with an initial potential in the 2.8–3.0 V vs. Na/Na^+^ range. Since all potentials in this study are referenced to the Na/Na^+^ redox pair, the term voltage is used instead of potential.

The electrochemical tests were conducted using a Biologic Battery Cycler BCS810 (Biologic Science Instruments, Seyssinet-Pariset, France). The two-electrode cells underwent galvanostatic cycling (discharge/charge cycles) between 2.1 and 0.01 V vs. Na/Na^+^ at a constant current density of 37.2 mA g^−1^ for 400 cycles. Cyclic voltammograms (CV) were recorded over the 2.1–0.01 V potential range vs. Na/Na^+^, at different scan rates, for 5 cycles.

## 4. Conclusions

Sustainable carbon materials derived from sucrose through a simple, fast, and cost-effective two-step eco-friendly process have been successfully applied as anodes in sodium-ion batteries. Among them, SC900 material featuring a partially developed graphite-like structure, though not strictly graphitic, showed the best electrochemical performance. The SC900-based electrode delivered discharge capacities exceeding 100 mAh g^−1^ after 400 discharge/charge cycles, with excellent cycling stability (99% capacity recovery) and coulombic efficiency (99%).

The extent of the Na^+^ ion electrochemical storage in the sucrose-based carbon materials is closely linked to their structural order, as indicated by the graphene interlayer distance, d_002_, from XRD and the relative intensity of the Raman D-band, I_D_/I_t_. In this context, the results show that the capacity provided by these materials increases as d_002_ decreases, reflecting a higher degree of graphite-like structural order, until reaching an optimal value of approximately 0.3700 nm. However, a further reduction in d_002_ to quasi-graphitic levels (~0.34 nm) in iron-doped sucrose-based carbons, as a consequence of the catalytic effect of iron, hinders Na^+^ ion storage, which combined with the low electrochemical activity of the iron oxides present leads to significantly lower capacities.

## Figures and Tables

**Table 1:** Elemental analysis (dry basis) of sucrose-based carbon materials.

Material	C (wt.%)	H (wt.%)	N (wt.%)	S (wt.%)	O (wt.%)	Fe (wt.%)
SC500	84.20	3.14	0.27	0.01	12.29	--
SC800	94.96	0.87	0.45	0.01	1.97	--
SC900	95.90	0.96	0.82	0.01	2.76	--
2FeSC900	95.18	0.64	0.45	0.01	2.13	2.20
9FeSC900	89.92	0.28	0.31	0.01	1.07	8.80

**Table 2:** Textural parameters of sucrose-based carbon materials: BET surface area (S_BET_), mesopore volume (V_meso_), micropore volume (V_micro_), mean pore size (D_p_), and external surface area (S_ext_).

Material	S_BET_ (m^2^ g^−1^)	V_meso_ (cm^3^ g^−1^)	V_micro_ (cm^3^ g^−1^)	D_p_ (nm)	S_ext_ (m^2^ g^−1^)
SC500	--	--	--	--	--
SC800	--	--	--	--	--
SC900	--	--	--	--	--
2FeSC900	163	0.097	0.059	5	114
9FeSC900	195	0.169	0.079	5	164

**Table 3:** Specific discharge capacity (C_disc_, mAh g^−1^) at the 1st, 2nd, 25th, 100th, 200th, and 400th cycles, irreversible capacity in the 1st cycle (C_irr_, %), and capacity retention between the 25 and 400th cycles (R, %) obtained from galvanostatic cycling vs. Na/Na^+^ at 37.2 mA g^−1^ for SC- and FeSC-based electrodes.

Electrode	C_disc_ 1st Cycle	C_disc_ 2nd Cycle	C_disc_ 25th Cycle	C_disc_ 100th Cycle	C_DISC_ 200th Cycle	C_DISC_ 400th Cycle	C_irr_ ^1^ 1st Cycle	R ^2^ 25–400th Cycles
SC500	118	45	81	88	84	80	78	93
SC800	270	112	104	105	102	96	64	94
SC900	145	173	107	92	110	111	77	99
2FeSC900	191	55	47	43	42	40	80	91
9FeSC900	284	68	48	43	42	39	80	90

^1^ Irreversible capacity (%) = [C_disc_ (1st cycle)—C_charge_ (1st cycle)] [C_disc_ (1st cycle)]^−1^ × 100. ^2^ Capacity retention (%) = [C_disc_ (400th cycle)] [C_disc_ (25th cycle)]^−1^ × 100.

## Data Availability

The original contributions of the study are included in the article; further inquiries can be directed to the corresponding authors.

## Supplementary Materials

The following supporting information can be downloaded at: https://www.mdpi.com/article/10.3390/molecules30051003/s1, Figure S1: Nitrogen adsorption–desorption isotherms of SC900, 2FeSC900, and 9FeSC900 sucrose-based carbon materials; Figure S2: Potential profiles (vs. Na/Na^+^) of SC and FeSC sucrose-based carbon materials from cycling at 37.2 mA g^−1^ in cycles 1, 2, 25, 50, 100, 200, and 400.
