# Supplementary material for: Sustainable Carbon Materials from Sucrose as Anodes for Sodium-Ion Batteries
Source: Molecules. 2025 Feb 21;30(5):1003. doi: 10.3390/molecules30051003 (PMC11901556; doi:10.3390/molecules30051003)
Supplement: Supplementary file 1 [file molecules-30-01003-s001.zip › molecules-3450411-supplementary.pdf]

molecules

MDPI

Article

Sustainable Carbon Materials from Sucrose as Anodes for Sodium-Ion Batteries

Belén Lobato*, Nuria Cuesta, Ignacio Cameán, M. Rosa Martínez-Tarazona*, Roberto García, Ana Arenillas and Ana B. García

Instituto de Ciencia y Tecnología del Carbono, INCAR-CSIC, Francisco Pintado Fe 26, 33011 Oviedo, Spain. belen@incar.csic.es (B.L.); n.cuesta@incar.csic.es (N.C.); rmtarazona@incar.csic.es (M.R.M.-T.); robo@incar.csic.es (R.G.); aapuente@incar.csic.es (A.A.); anabgs@incar.csic.es (A.B.G.)

- Correspondence: rmtarazona@incar.csic.es (M.R.M.-T.); belen@incar.csic.es (B.L.)

SUPPLEMENTARY MATERIAL

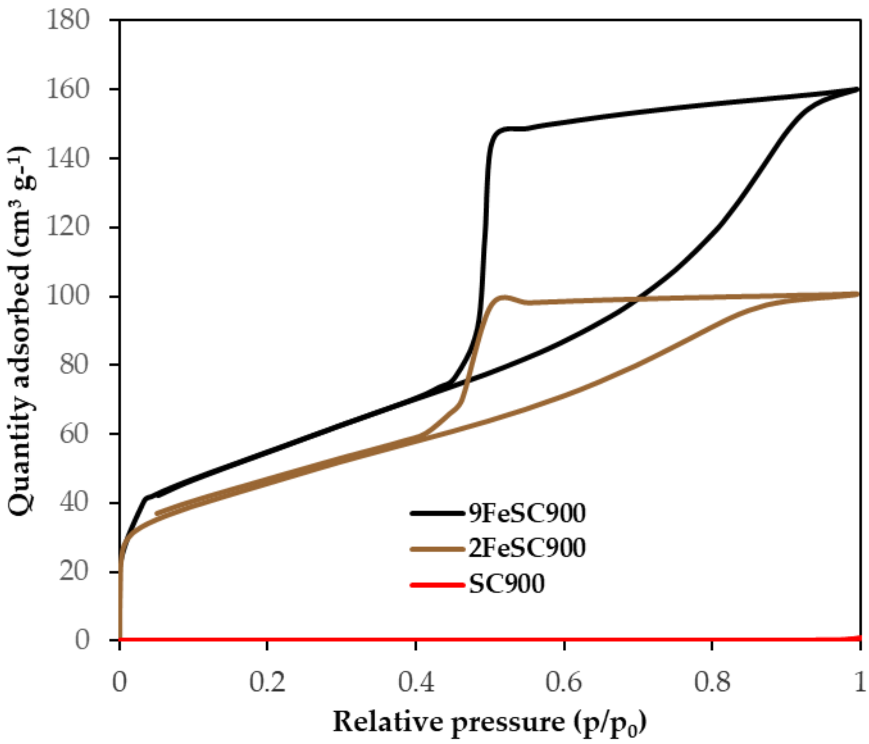

Figure S1. Nitrogen adsorption-desorption isotherms of SC900, 2FeSC900 and 9FeSC900 sucrose-based carbon materials.

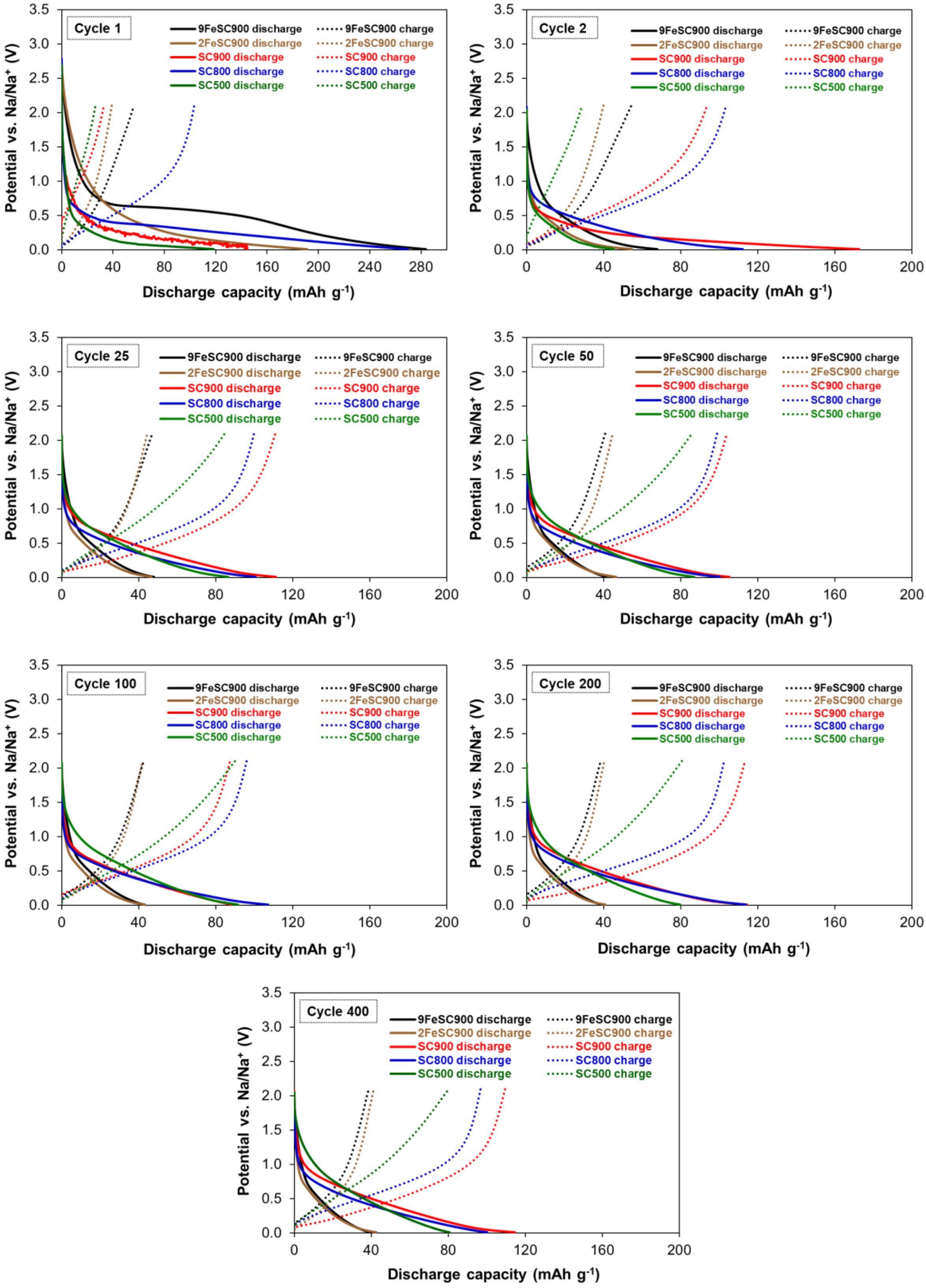

Figure S2. Potential profiles (*vs.* Na/Na$^+$) of SC and FeSC sucrose-based carbon materials from the cycling at 37.2 mA g^{-1} in cycles 1, 2, 25, 50, 100, 200 and 400.
